# Acute Appendicitis Management in Patients Aged Above 40 Years During the COVID-19 Pandemic: A Retrospective Study With Four Years of Follow-Up

**DOI:** 10.7759/cureus.73196

**Published:** 2024-11-07

**Authors:** Rahel Rashid, Mohamed Y Abuahmed, Baidar Khalabazyane, Kamalesh Inteti, Israa Kadhmawi, Ahmed Awakhti, Jeremy Wilson, Conor Magee

**Affiliations:** 1 General and Colorectal Surgery, Arrowe Park Hospital, Wirral, GBR; 2 Upper Gastrointestinal Surgery, Wirral University Teaching Hospital NHS Foundation Trust, Wirral, GBR; 3 Urology, Royal Bournemouth Hospital, Bournemouth, GBR; 4 General Surgery, Shar Teaching Hospital, Sulaymaniyah, IRQ; 5 General Surgery, Wirral University Teaching Hospital NHS Foundation Trust, Wirral, GBR

**Keywords:** acute appendicitis, conservative management of acute appendicitis, covid-19, elective appendicectomy, nom acute appendicitis

## Abstract

Background

The COVID-19 pandemic led many units to increase their utilization of nonoperative management (NOM) of acute appendicitis, with the literature showing its non-inferiority when compared to operative management (OM). Therefore, we compared NOM to OM against standard guidelines in order to ascertain the effectiveness of NOM. Primary outcomes were rates of admission, complications, hospital length of stay (LOS), and the incidence of colonic malignancy following NOM upon subsequent bowel evaluation using colonoscopy and/or computed tomography (CT) scan.

Methods

This was a retrospective observational study done on patients who were admitted with acute appendicitis from January 2020 to January 2022 at Wirral University Teaching Hospital, UK. Data was electronically collected from medical records. Inclusion criteria were patients aged 40 years and above, admitted with a diagnosis of acute appendicitis using a CT scan, and who underwent either OM or NOM. Exclusion criteria were patients below 40 years old or not diagnosed with acute appendicitis.

Results

This study included 211 cases of acute appendicitis (female to male: 110:101), with a median age of 60. One hundred and twenty-five (60%) patients were managed operatively, while 86 cases (40%) were managed by NOM. All of the cases were diagnosed using a CT scan. The mean LOS for operative and non-operative cases were 4.77 and 4.89 days, respectively. When readmission days were added over the following three years, adjusted LOS was 5.35 days for operative cases, in comparison to 10.86 days for NOM. Forty-five percent of NOM cases had at least one episode of readmission, with 37% of them being in the first year. We found six cases of malignancy in the NOM cohort, none of which were detected on colonoscopy following discharge.

Conclusion

NOM is associated with increased LOS and increased readmission rates, and 44% of cases eventually required appendicectomy.

## Introduction

Acute appendicitis is the most prevalent general surgical emergency worldwide [1]. Since the advent of appendicectomy in the 19th century, surgery has been the most widely accepted treatment for appendicitis [2], with around 40,000 appendicectomies performed in the UK annually [3]. Current evidence indicates that laparoscopic appendicectomy (LA) is the superior surgical treatment, demonstrating shorter hospital stays and a lower incidence of post-intervention morbidity and wound infections in comparison to open appendectomy (OA).

The nonoperative management (NOM) of acute appendicitis has been discussed in the literature, with renewed interest more recently, where in the era of COVID-19, NOM has emerged as a significant alternative to surgery for uncomplicated acute appendicitis cases.

The COVID-19 pandemic placed significant strain on NHS resources in the UK. There was a persistent shortage of personal protective equipment (PPE), along with concerns about infection spread during aerosol-generating procedures (AGPs), such as surgeries, specifically laparoscopic approaches. Additionally, evidence indicated increased general and pulmonary complications in COVID-19-positive patients. As a result, the NOM of acute appendicitis was endorsed by the 2020 World Society of Emergency Surgery (WSES) guidelines [4], the CODA trial, which showed the non-inferiority of NOM [5], as well as the royal colleges of surgeons in the UK and various other well-recognized authorities [6,7].

The nonoperative or conservative management of acute appendicitis typically involves a trial of administering intravenous (IV) antibiotics, with subsequent conversion to oral antibiotics, and, if required, utilizing interventional radiology (IR) drainage for associated collections or abscesses [4]. This mode of management has a reported recurrence rate of more than 20% in one year after the first episode and up to 39% after five years [8-10].

Among adult patients aged 40 years and older who present with complicated appendicitis, the incidence of appendicular neoplasms ranges from 3% to 17% [4], with more cases reported in the conservatively managed cohort [11,12]. Hence, the WSES advocates screening and bowel evaluation with both computed tomography (CT) scan and colonoscopy.

At our center, we observed a rising trend of NOM for acute appendicitis during the COVID-19 pandemic, which was the case worldwide as well. Therefore, we decided to review our findings with the added benefit of a four-year follow-up. We aimed to ascertain the effectiveness of NOM in treating acute appendicitis, the hospital length of stay (LOS), the efficacy and utilization of bowel screening, and the readmission rates after discharge in comparison to the operatively managed cohort from the same time frame.

## Materials and methods

This was a retrospective observational study conducted at Arrowe Park Hospital in the UK. Data were collected through the use of electronic health records (EHR) in the Cerner Millennium software, a platform that provides a comprehensive view of patient care across the enterprise, capturing information at the point of care delivery for all adult patients aged ≥40 years who were diagnosed with acute appendicitis from January 2020 to January 2022 regardless of management type. Inclusion criteria were as follows: (1) diagnosis of acute appendicitis (radiologically using CT scan) and (2) patients aged ≥40 years. In contrast, patients younger than 40 years, patients with no (radiological) diagnosis of acute appendicitis, and known colon cancer patients were excluded from the study. Statistical analysis was performed using jamovi (Version 2.5) (the jamovi project (2023); retrieved from https://www.jamovi.org). Student's t-test was used for quantitative data and a p-value of <0.05 was considered to be statistically significant.

The collected data included age, gender, presenting symptoms, the utilization of scoring systems, COVID-19 infection status, imaging studies, hospital LOS, and type of management (operative or conservative). Patients were then categorized depending on their management for further data gathering. In the operated cohort, further data were collected on time to operation from admission, the use of LA vs OA, the use of peritoneal irrigation or suction, the use of intra-abdominal drains, the incidence of intra-abdominal collections, surgical site infection (SSI), and other complications.

**Figure 1:**
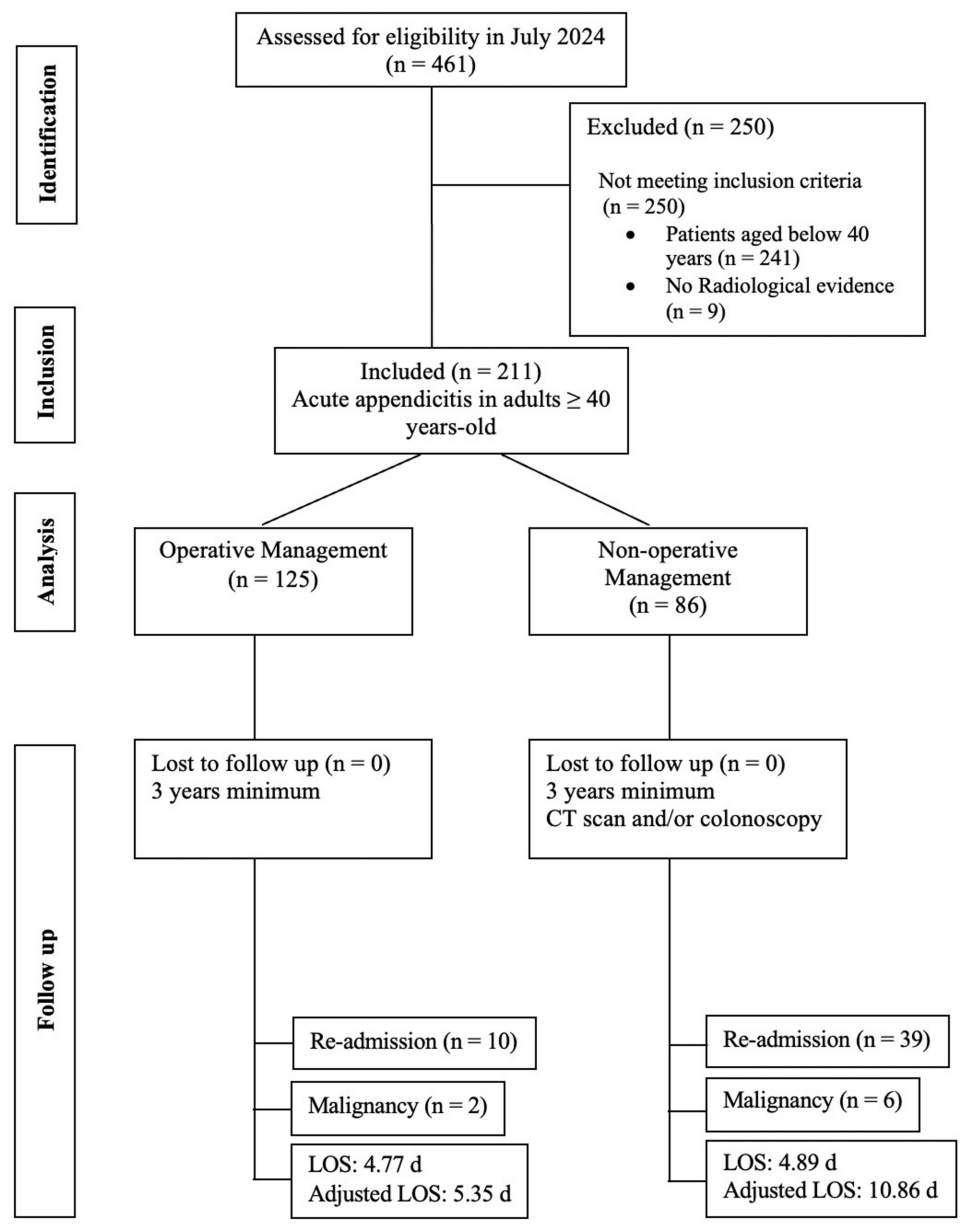
STROBE diagram showing the study design and exclusion criteria n: number; LOS: length of stay; CT: computed tomography; STROBE: STrengthening the Reporting of OBservational studies in Epidemiology

As for the conservatively managed cohort, data included rates of interval appendicectomy, the incidence and rate of recurrence and re-admissions, and the frequency of follow-up as well as bowel evaluation. The methodology is shown in Figure 1.

## Results

Over the two-year period, our hospital recorded 461 admissions for acute appendicitis. Exclusion criteria were applied: 241 patients aged <40 years and no radiological evidence of acute appendicitis in nine cases. The remaining 211 cases that fulfilled our inclusion criteria were subsequently analyzed. Of these, 52% (n=110) were female, and 48% (n=101) were male, with a median age of 60 years (ranging from 40 to 93 years). The vast majority, 97.6% (n=206), were admitted on an emergency basis, while only five cases were admitted electively. Approximately 59% (n=125) of the cases were managed operatively, while 41% were treated conservatively.

Regarding COVID-19 status, 189 (89.6%) cases underwent nasal swabs, seven (3.3%) cases received CT of the thorax, and 15 (7.1%) cases did not undergo any testing. Seven patients (3.4%) tested positive for the virus via nasal swab polymerase chain reaction (PCR), and 189 (89.6%) cases tested negative. Among the COVID-19-positive individuals, one patient (14%) developed a pulmonary embolism postoperatively.

Our findings revealed that none of the cases underwent any scoring system, such as the Alvarado or Appendicitis Inflammatory Response (AIR) score, during their diagnostic evaluation. All patients received a CT scan. 

**Figure 2:**
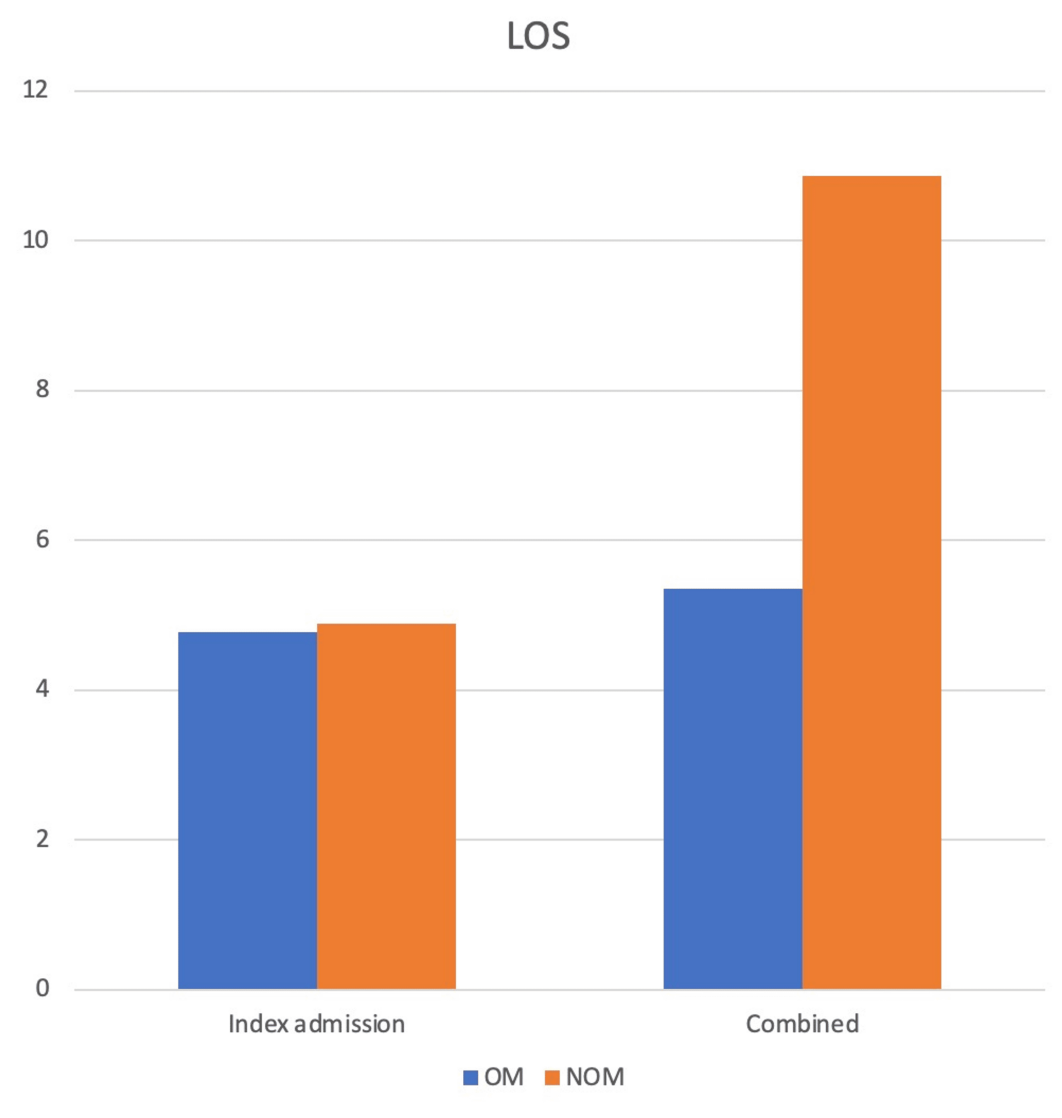
Bar chart showing the difference in LOS between both groups and the combined LOS LOS: length of stay; OM: operative management; NOM: nonoperative management

To assess the hospital LOS, we initially calculated the LOS during the index admission and then incorporated the additional LOS from recurrent episodes and readmissions. The overall mean LOS for the entire study sample at the index admission was 5.82 days, with operative management (OM) and NOM averaging 4.77 days and 4.89 days, respectively. However, when accounting for readmissions, 10 out of 125 cases (8%) in the operative group were readmitted, contributing an additional 16 days to the total LOS. In contrast, the NOM cohort experienced 39 readmissions, resulting in an extra 359 days of LOS. After adjusting for these additional days, the mean adjusted LOS for the operative group was 5.35 days, compared to 10.86 days for the NOM group. The LOS is shown in Figure 2.

OM

Out of the 125 cases that underwent emergency appendicectomy, all were performed using a laparoscopic approach, with 3.2% (n=4) requiring conversion to an open procedure. The median time from admission to operation was 18 hours (range 3-96 hours). Peritoneal irrigation with normal saline was employed in 94% (n=117) of the cases, while none utilized a suction-only method for managing peritoneal soiling, and eight (6.4%) patients did not receive any irrigation. Intra-abdominal drains were placed in 31% (n=39) of the cases. The incidence of intra-abdominal abscesses (IAA) was 11%, and no cases of SSI were reported in this group.

Within this cohort, two cases of appendicular neoplasm were identified. The first was a mucinous appendicular neoplasm discovered through histopathological examination postoperatively, which led to the detection of peritoneal metastases on a follow-up CT scan six months later. The patient was subsequently referred to a regional specialist center, where they underwent successful cytoreductive surgery combined with hyperthermic intraperitoneal chemotherapy (HIPEC). The second case involved a diagnosis of appendiceal adenocarcinoma on admission, which was successfully treated with a right hemicolectomy and ileocolostomy.

Two cases had postoperative mortality. The first was a 93-year-old patient who developed heart failure, and the second was a 71-year-old patient who developed ileus. 

A number of other complications following appendicectomy were documented, which are summarized in Table 1.

**Table 1:** Complications following laparoscopic appendicectomy NOM: nonoperative management

Complication	Number (%)
Conversion to open	4 (3.2)
Cecal injury	1 (0.8)
Port-site hernia	2 (1.6)
Stump appendicitis	2 (1.6)
Failed NOM	5 (4)
Critical care admission	3 (2.4)
Postoperative pneumonia	2 (1.6)
Ileus	5 (4)
Pulmonary embolism	1 (0.8)

NOM

This group included 86 patients, of which 35 (40.7%) were male and 51 (59.3%) were female, with a median age of 60 years. Interval appendicectomy was performed in 43% (n=37) of cases. Of these cases, 12 were elective procedures, while 25 were emergency interventions, due to either failed NOM or recurrent acute presentation of appendicitis previously managed conservatively. 

An additional case involved a palliative ileocecal bypass due to a locally advanced appendiceal/cecal malignancy. This patient initially presented six months earlier with an appendiceal abscess that responded to IV antibiotics and drainage. Eight weeks post-presentation, a colonoscopy revealed only benign cecal polyps, with no signs of malignancy. Despite multiple CT scans during recurrent admissions, a complex mass was eventually detected five months later. Intraoperatively, the mass was found to have invaded the external iliac artery, rendering it unresectable. The patient passed away 10 months after the initial episode.

Regarding bowel evaluation and screening, it was conducted in 50% (n=43) of the cases. Among these, 21 (48.8%) cases received both a CT scan and colonoscopy, while nine (20.9%) cases underwent only a CT scan, and 13 (30.2%) cases had a colonoscopy alone. None of the cases that underwent CT scans or colonoscopies revealed any malignant pathology. In the remaining 50% of NOM cases, no bowel screening or evaluation was offered. For 11 (25.5%) of these cases, the decision was based on clinical circumstances, such as the patient's mortality or a previous colonoscopy a few years prior. Six cases of appendiceal neoplasms were documented (p=0.045). The first case, described in detail earlier, involved a palliative ileocecal bypass (no histopathology available). The second case was a neuroendocrine tumor (NET) detected following interval appendicectomy for recurrent acute appendicitis three years after the initial episode; histopathology revealed a 2.7 mm NET. The third case involved a mucinous appendiceal neoplasm diagnosed after a recurrent episode of acute appendicitis, occurring 10 days after discharge from the initial episode. This patient also underwent an appendicectomy but, unfortunately, passed away 12 months after the initial presentation.

The fourth case experienced a recurrent episode of appendicitis one month following the initial admission. A colonoscopy was performed five months later, which did not reveal any significant pathology. An interval LA was scheduled but had to be converted to an open ileocecectomy. Histopathological examination revealed a mucinous neoplasm of the appendix. Postoperatively, the patient developed an anastomotic leak, which led to an SSI and wound dehiscence. Notably, this was the only case of SSI recorded in the entire study sample.

The fifth case involved a 59-year-old patient who presented with a concerning mass in the right iliac fossa, as detected on a CT scan. The patient was initially managed conservatively and underwent additional CT scans and a colonoscopy. A planned right hemicolectomy was subsequently performed, and histopathological analysis confirmed the presence of appendiceal adenocarcinoma.

The sixth case involved a 67-year-old patient whose initial CT scan indicated uncomplicated acute appendicitis, leading to conservative management. A subsequent colonoscopy revealed hyperplastic polyps. The patient was readmitted nine months later, at which point an emergency interval appendicectomy was performed. Histopathological examination of the specimen revealed a mucinous appendiceal neoplasm.

**Figure 3:**
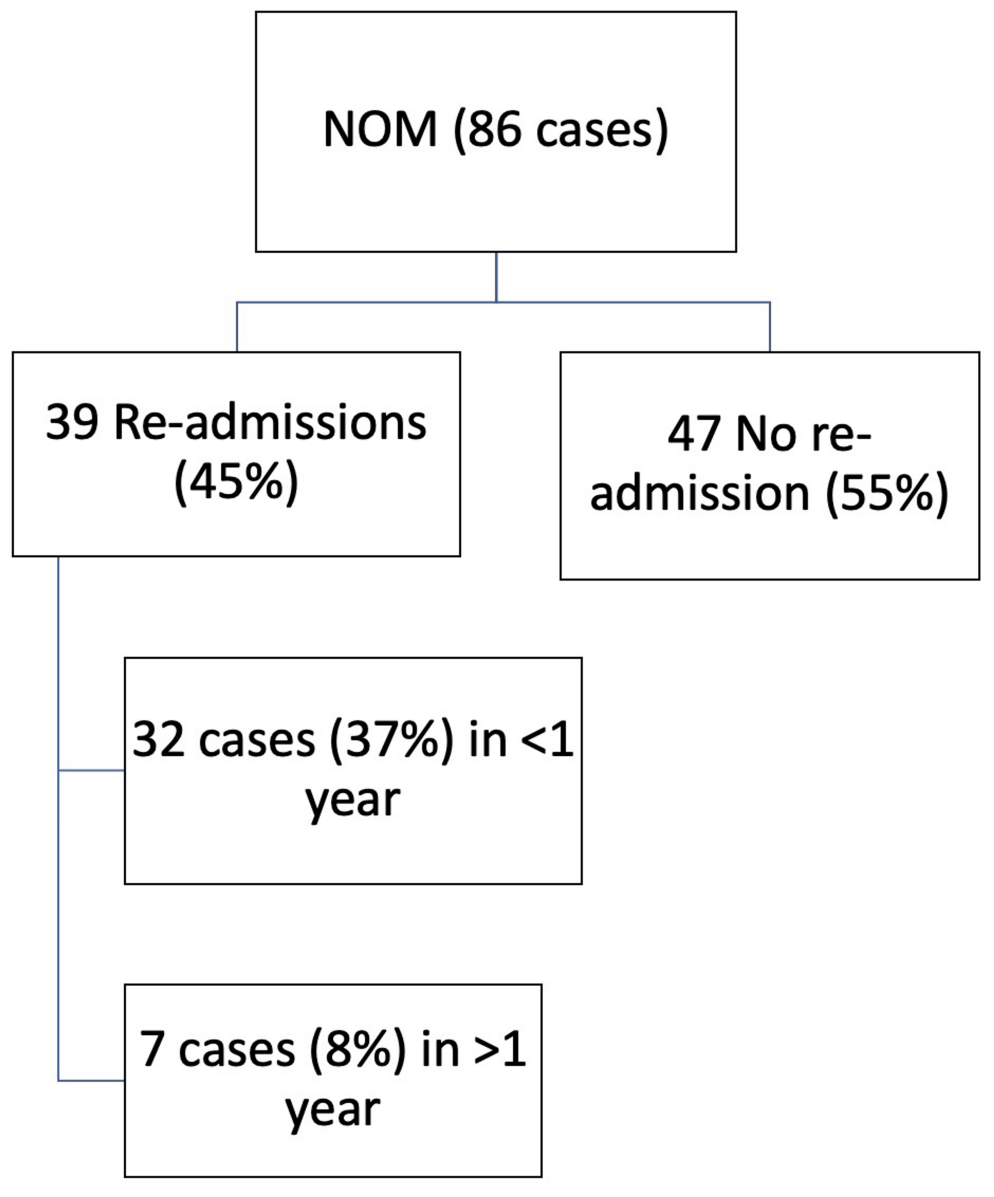
A flowchart showing readmission rates among the NOM group NOM: nonoperative management

Regarding readmission rates, we found that 45% (39 out of 86 patients) of NOM cases were readmitted at some point following an initial episode of acute appendicitis. Thirty-seven percent (32 out of 86 patients) were readmitted within 12 months, and seven cases (8%) were readmitted after one year, making a combined readmission rate of 45% in less than four years. This can be shown in Figure 3.

IAA

This outcome was assessed across all cases that underwent surgery, whether initially OM or following NOM. In total, 20 cases developed IAA, with an incidence of 12% (15 out of 125) in the OM cohort and 13.5% (five out of 37) in the NOM cohort that eventually required an appendicectomy. All 20 cases had received peritoneal irrigation, and 50% (10 out of 20) had an intra-abdominal drain placed (p=0.151).

## Discussion

This study aimed to assess the long-term outcomes of NOM of acute appendicitis compared to OM. Due to the COVID-19 pandemic, many authorities recommended a conservative approach to treating acute appendicitis. While several interim analyses were published during the pandemic [4-7], this study focuses on evaluating patient outcomes four years after conservative treatment.

Impact of COVID-19 on patient volume

Over the two-year study period, we observed around 105 admissions per year, consistent with local data from previous and following years. This contrasts with Rosenthal et al.'s study [13], which reported a significant drop in patient volume during the COVID-19 pandemic. In our cohort, 41% of cases were managed conservatively, reflecting an increasing trend in NOM, similar to Emile et al.'s systematic review, which reported a 44% application rate for NOM [14].

Outcomes of COVID-19

In our cohort, 93% of cases underwent COVID-19 testing, mostly via nasal swabs (89.6%), with only seven cases receiving CT of the thorax. Testing occurred during the early pandemic, before formal protocols were in place, explaining why the first 15 cases were not tested. Among the COVID-19-positive cases, only one developed a pulmonary embolism, while the others experienced no pulmonary or other complications, regardless of the treatment method. This contrasts with earlier reports suggesting worse postoperative outcomes for COVID-19-positive patients [15].

Clinical scoring systems

Clinical scoring systems are essential for improving decision-making in patients with suspected acute appendicitis, as they help reduce unnecessary admissions and optimize diagnostic imaging use. These tools are particularly useful in identifying low-risk patients, minimizing the need for imaging, and avoiding unnecessary surgeries [4]. A study by Bhangu and the Right Iliac Fossa Pain Treatment (RIFT) Study Group, involving 5,345 patients across 154 UK hospitals, evaluated 15 risk models. The Adult Appendicitis Score (AAS) was most effective for women, while the AIR score was most reliable for men, with varying specificities and failure rates [16].

Imaging

All of our patients were offered a CT scan (abdomen and pelvis), differing from the WSES recommendation, which highlights the value of point-of-care ultrasonography (POCUS) for diagnosing acute appendicitis. POCUS, especially when combined with a clinical scoring system, can enhance decision-making. While ultrasound (US) has a sensitivity of 76% and a specificity of 95%, CT scans provide higher sensitivity (99%) but lower specificity (84%) [4].

Hospital LOS

We observed similar hospital LOS for both OM and NOM cases during their initial admission. However, when adjusting for recurrent episodes and additional readmissions, NOM resulted in nearly double the LOS (10.86 days) compared to OM (5.35 days). This finding contrasts with the Jerusalem Guidelines by WSES and Helling et al. [4,17], but aligns with other literature reports [18,19].

OM

Of the 125 patients (59%) who underwent operative management, the median time to surgery was 18 hours, well within the recommended 24-hour window [4]. We identified 20 (12.3%) cases of IAA, all of which had received peritoneal irrigation with normal saline, with 10 (50%) of these cases also receiving abdominal drains. Conversely, eight patients who did not receive peritoneal irrigation did not develop IAA. This discrepancy may be attributed to the selective nature of the pathology; complicated appendicitis, such as perforated acute appendicitis, is an independent risk factor for developing IAA postoperatively [20] and is more likely to receive peritoneal irrigation. Current evidence suggests that peritoneal irrigation with normal saline during LA does not significantly reduce the incidence of IAA, SSI, or hospital LOS compared to suction alone, although it may prolong the operation [4].

NOM

In our study, 86 patients underwent NOM, accounting for 41% of the total sample. Literature indicates a recurrence rate of up to 20% within one year and up to 39% within five years for NOM cases [8-10]. In our cohort, the recurrence rate was 37% within the first year and 45% over four years of follow-up, potentially increasing further if the follow-up period was extended to indicate five years. Additionally, NOM cases had an average total LOS approximately twice as long as that of operatively managed cases. The literature suggests bowel screening and follow-up with both CT scans and colonoscopy [4], as appendicular neoplasms are more frequently observed in conservatively managed acute appendicitis cases [11,12,21]. It is essential to note that we identified six cases of appendiceal neoplasm, none of which were detected through colonoscopy. According to the WSES guidelines, routine interval appendectomy is not recommended for patients under 40 years of age who are managed nonoperatively unless they exhibit recurrent symptoms. Our institution's practice closely aligns with this guideline, offering routine interval appendectomy to only 12 patients. Additionally, 25 patients underwent emergency interval appendectomy due to recurrent acute appendicitis. Of the 37 patients who received interval appendectomy, whether elective or emergency, six cases (16.2%) were found to be malignant appendiceal neoplasms, comprising nearly 7% of total NOM cases (6/86). This is very similar to de Jonge et al. who found that in adult patients with complicated acute appendicitis treated with interval appendectomy, the incidence of appendiceal neoplasm can be as high as 11%, compared to just 1.5% in those who undergo early appendectomy [11,22]. Similarly, the randomized controlled trial (RCT) conducted by Mällinen et al., which compared interval appendectomy with follow-up using magnetic resonance imaging (MRI) after initial successful nonoperative treatment of periappendicular abscess, was prematurely terminated due to ethical concerns. This decision was prompted by an unexpected finding during the interim analysis, which revealed a high incidence of neoplasms (17%), all of which were found in patients over 40 years of age [23].

Strengths and limitations

This study provides a four-year follow-up of patients with acute appendicitis who were managed non-operatively during the COVID-19 pandemic. It offers valuable insights into the outcomes of NOM, the incidence of appendicular neoplasms, and the effectiveness of bowel screening. Limitations of the study include its single-center design, which focused exclusively on adult patients, the absence of randomization, and a follow-up period that may be shorter than ideal.

## Conclusions

Our findings show that while NOM was initially effective, it led to higher readmission rates, longer hospital stays, and a 7% occurrence of appendiceal neoplasms, none detected by routine screening. With a 45% recurrence rate over four years, NOM's long-term risks are more significant than previously reported. These results suggest that NOM should be used cautiously, especially in older adults, as it raises concerns about cost-effectiveness and long-term outcomes. Future research should improve patient selection criteria, follow-up protocols, and screening for appendiceal neoplasms in NOM cases.
